# Fermented foods, microbiota, and mental health: ancient practice meets nutritional psychiatry

**DOI:** 10.1186/1880-6805-33-2

**Published:** 2014-01-15

**Authors:** Eva M Selhub, Alan C Logan, Alison C Bested

**Affiliations:** 1 Harvard Medical School and Massachusetts General Hospital, 40 Crescent St., Suite 201, Waltham, MA 02453, USA; 2 CAMNR, 23679 Calabasas Road Suite 542, Calabasas, CA 91302, USA; 3 Complex Chronic Diseases Program, BC Women’s Hospital and Health Centre, B223A-4500 Oak Street, Vancouver, BC V6H 3N1, Canada

## Abstract

The purposeful application of fermentation in food and beverage preparation, as a means to provide palatability, nutritional value, preservative, and medicinal properties, is an ancient practice. Fermented foods and beverages continue to make a significant contribution to the overall patterns of traditional dietary practices. As our knowledge of the human microbiome increases, including its connection to mental health (for example, anxiety and depression), it is becoming increasingly clear that there are untold connections between our resident microbes and many aspects of physiology. Of relevance to this research are new findings concerning the ways in which fermentation alters dietary items pre-consumption, and in turn, the ways in which fermentation-enriched chemicals (for example, lactoferrin, bioactive peptides) and newly formed phytochemicals (for example, unique flavonoids) may act upon our own intestinal microbiota profile. Here, we argue that the consumption of fermented foods may be particularly relevant to the emerging research linking traditional dietary practices and positive mental health. The extent to which traditional dietary items may mitigate inflammation and oxidative stress may be controlled, at least to some degree, by microbiota. It is our contention that properly controlled fermentation may often amplify the specific nutrient and phytochemical content of foods, the ultimate value of which may associated with mental health; furthermore, we also argue that the microbes (for example, *Lactobacillus* and *Bifidobacteria* species) associated with fermented foods may also influence brain health via direct and indirect pathways.

## Introduction

‘*The processes required for fermented foods were present on earth when man appeared on the scene… When we study these foods, we are in fact studying the most intimate relationships between man, microbe and foods.*’ [1]

Prof. Keith H. Steinkraus, Cornell University, 1993

As highlighted in the quotation, our Paleolithic ancestors had plenty of opportunity for the consumption of food products (for example, honey, fruits or berries, and their juices) that had been unknowingly subjected to natural microbial fermentation. Without knowledge of microbes, our ancestors recognized, over time, the palatability, preservative, analgesic, and mentally stimulating or sedating qualities of fermented foods and beverages [2]. Thus, the stage was set for the purposeful application of fermentation to provide value in the areas of human nutrition, traditional medicine, and culture (ceremonies, and so on) [3,4]. It is difficult to say with certainty when intentional fermentation began in earnest; however, sophisticated measurements of the chemical content within ancient Neolithic vessels suggest intentional fermentation of fruit, rice, or honey beverages has been in common practice for close to 10,000 years [5]. As agriculture expanded, so too did intentional fermentation techniques. Beyond the clear references to alcohol production, it is now obvious that household and artisanal fermentation of cereals, dairy, vegetables, fish, seafood and meats were a significant part of ancestral dietary practices [6].

Modern advances in chemical preservation, refrigeration, and transportation efficiency have not resulted in the abandonment of fermented foods. At least in traditional dietary practices, fermented foods and beverages remain widespread, currently accounting for approximately one-third of the human diet globally [7]. Moreover, as scientists continue to uncover health-promoting properties of ancestral dietary patterns (for example, the Mediterranean diet, the traditional Japanese diet, and hunter-gatherer diets), by extension there is a renewed examination of the fermented foods that are so often a part of such ancient diets [8]. Emerging research, as reviewed here, indicates that fermentation may magnify the known benefits of a wide variety of foods and herbs, influencing the bioavailability and activity of the chemical constituents. In addition, as our knowledge of the human microbiome increases (the intestinal microbiota in particular), it is becoming increasingly clear that there are untold connections between the ways in which microbes act upon dietary items pre-consumption, and in turn, the ways in which these fermented dietary items influence our own microbiota.

Here, we review and synthesize various lines of investigation related to fermented foods, intestinal microbiota, and mental outlook. We argue that the consumption of fermented foods may be particularly relevant to the emerging research linking traditional dietary practices and positive mental health. It is our contention that fermentation may amplify the specific nutrient or phytonutrient content of foods, the ultimate value of which is associated with mental health; furthermore, we also argue that the microbes associated with fermented foods may also influence brain health via direct and indirect pathways.

## Traditional diets and mental health

The shift away from traditional lifestyles has been linked to increased rates of depression and other mental health disorders [9-11]. Among the variables that might afford protective or resiliency effects against mental health disorders (depression in particular), diet has emerged as at least one strong candidate [12]. Superficially, it would seem obvious, given the brain’s dependence upon nutrients for its structure and function (including the micronutrients and non-nutrient dietary antioxidants, for example polyphenols, that run the antioxidant defense system) that nutrition should be a target of research in mental health. Remarkably, this area of research, now known as nutritional psychiatry, is one that has been historically neglected or the subject of poorly designed studies. However, there have been tremendous strides in recent years and the research connecting mental health and nutrition has become increasingly robust. Indeed, a recent 5-year prospective study (*n*?=?23,020) has shown that unhealthy maternal and early postnatal dietary patterns (for example, processed and refined foods, high-sugar beverages, high-sodium snacks) elevate the risk of behavioral and emotional problems in children [13].

Traditional dietary practices, often exemplified by Mediterranean and Japanese models, are typically characterized by (relative to Western practices) higher intakes of fruits and vegetables, fish and seafood, cereals with limited processing, fiber, and only modest amounts of dairy and lean meats [14]. A variety of population studies have linked adherence to traditional dietary patterns with lowered risk of anxiety or depression [15-20]. Among the more convincing of these studies are the recent prospective investigations showing that stronger adherence to traditional healthy dietary patterns is associated with a 25 to 30% lower risk of depression [16,17]. Traditional Japanese dietary practices, where fermented soy products are specifically linked to adherence, have also been associated with lower rates of depressive symptoms [21,22]. Alcohol has deservedly received much attention in the link between problematic consumption and a higher risk of depression. However, when consumed in modest amounts (5 to 15 g per day) as part of traditional dietary practices, alcohol (red wine in particular) has been associated with a lower risk of depression [23]. Indeed, light to moderate alcohol consumption has been associated with lower systemic inflammation, a finding not evident in those with depression [24].

Epidemiological studies show that there exists an elevated risk of depressive symptoms in healthy adults with blood chemistry indicative of insulin resistance [25]. Depressive symptoms correlate with higher fasting and stimulated glucose levels, even in the absence of an association with adiposity in adolescents at risk of type II diabetes [26]. This is of relevance when viewed along with a rapidly growing body of research highlighting the type-II-diabetes protective properties of traditional dietary practices [27]. As we will discuss, the intestinal microbiota, via a number of mechanisms, may play a role in mediating the glycemic and mood related effects of the Western dietary pattern [28].

Specific items within traditional dietary patterns have been individually associated with protection against depression and, experimentally, these components have also demonstrated antidepressant properties. Examples include, but are not limited to soy foods, turmeric, cocoa, green tea, coffee, blueberries, pomegranate, and honey. The isolated polyphenols and other phytochemicals within these foods have also been documented to provide antidepressive properties in experimental models [29-38]. In addition, specific nutrients such as magnesium, zinc, vitamin C, folic acid, and vitamin B12, have also been connected to resiliency against depression or improvement in depressive symptoms [39-42].

The mechanisms by which required nutrients, such as the aforementioned vitamins and minerals, influence mood can be explained in part by their role in the production of neurotransmitters [43]. However, the connection between mood and non-essential dietary components (for example, phytochemicals) has been the subject of intense scrutiny; their role in the antioxidant defense system as well as their ability to provide anti-inflammatory support appears to be at play [44]. Advances in the understanding of the pathophysiology of mood and anxiety disorders have provided a more complete picture of the inducing role played by the tandem of oxidative stress and low-grade inflammation. Elevations in markers of inflammation (for example, cytokines, C-reactive protein), and overwhelm of the normal antioxidant defense system, are no longer dismissed as mere consequence in emotional disorders [45]. The burden of oxidative stress and inflammation is emerging as a viscous cycle that can directly influence mood, and the combination of the two appears to be both a cause and a consequence of depression [46,47]. When levels of body-wide inflammatory cytokines are elevated, they can subsequently signal the production of inflammatory cytokines within the central nervous system via microglia activation. Chronic activation of microglia can compromise neuronal functioning by setting in motion a cascade of further inflammation and oxidative stress [48]. The end result may manifest as compromised intra and extracellular neuronal communication.

## Inflammation and mood pathways

In this exciting area of research, one of the open questions is how chronic inflammation might be initiated and maintained in illnesses such as depression, and what the gut has to do with this. Emerging studies show that the normally very selective intestinal barrier may be compromised in depression (and in numerous conditions where depression is often a hallmark symptom) [49-56]. Psychological stress and exhaustive exercise have been shown to increase the permeability of the intestinal barrier [57-59]. However, a Westernized diet high in fat and sugar has also been shown to cause a more porous intestinal lining, the consequences of which include systemic access to food antigens, environmental toxins, and structural components of microbes, such as lipopolysaccharide endotoxin (LPS) [60]. The latter agent, LPS, is particularly important regarding depression; even relatively small elevations in systemic LPS levels have been shown to provoke depressive symptoms and disturb blood glucose control [61-67]. Endotoxins such as LPS can decrease the availability of tryptophan and zinc, thereby negatively influencing neurotransmission [68,69]. Moreover, systemic LPS can elevate inflammation and oxidative stress. Traditional dietary practices have completely divergent effects of blood LPS levels; significant reductions (38%) have been noted after a one-month adherence to a prudent (traditional) diet, while the Western diet provokes LPS elevations [70]. These and other findings help establish mechanisms whereby the LPS-lowering, antioxidant, and anti-inflammatory properties of broad traditional dietary practices, as well as specific components within them, can help provide mood support. Indeed, when the limitation of intestinal absorption is overcome, individual phenolic structures have been shown, at least experimentally, to curb the breakdown of central neurotransmitters, mimicking the proposed mechanistic properties of some primary antidepressant medications [71,72]. As we will discuss, enhanced bioavailability via fermentation may therefore be an important factor in food (or herbs) as medicine.

## Microbiota and mental health

Related to the differences in traditional versus contemporary Westernized dietary patterns and mental health is the role of the intestinal microbiota. A decade ago, prior to the scientific hypotheses of Logan *et al.*[73,74], the notion that the intentional manipulation of the intestinal microbiota could provide therapeutic value to human depressive and fatigue states was, at the very least, outlandish. However, in the ensuing years, many of the mechanisms first proposed by Logan and colleagues (as listed, adapted from [73,74]) whereby beneficial microbes could influence mood or fatigue, have been examined experimentally.

•Direct protection of the intestinal barrier;

•Influence on local and systemic antioxidant status, reduction in lipid peroxidation;

•Direct, microbial-produced neurochemical production, for example, gamma-aminobutyric acid (GABA);

•Indirect influence on neurotransmitter or neuropeptide production;

•Prevention of stress-induced alterations to overall intestinal microbiota;

•Direct activation of neural pathways between gut and brain;

•Limitation of inflammatory cytokine production;

•Modulation of neurotrophic chemicals, including brain-derived neurotrophic factor;

•Limitation of carbohydrate malabsorption;

•Improvement of nutritional status, for example, omega-3 fatty acids, minerals, dietary phytochemicals;

•Limitation of small intestinal bacterial overgrowth;

•Reduction of amine or uremic toxin burden;

•Limitation of gastric or intestinal pathogens (for example, *Helicobacter pylori*);

•Analgesic properties.

Moreover, preliminary placebo-controlled human studies have shown that oral probiotic microbes can decrease anxiety, diminish perceptions of stress, and improve mental outlook [75]. In the context of our later discussion of fermented foods and their intersection with the gut-brain-microbiota connection, a brief summary of this microbiota-brain research is necessary. For interested readers, more detailed reviews specific to the scientific advances exploring direct and indirect relationships between intestinal microbes and anxiety or depression have recently been published [76,77].

Viewed strictly from the nutritional perspective, experimental studies have shown that the administration of probiotic bacteria to laboratory chow can increase peripheral tryptophan levels, and alter dopamine and serotonin turnover in the frontal cortex and limbic system [78]. In addition, probiotic-fortified laboratory chow increases the tissue levels of omega-3 fatty acids [79], and the omega-3 fatty acids play a critical role in communication in and between nerve cells. The consumption of omega-3 fatty acids, eicosapentaenoic acid in particular, has been linked to positive mental outlook and reduction in mental distress in human beings [80]. Levels of other anti-inflammatory fatty acids, such as gamma-linolenic acid, also increase in the human plasma when co-administered with probiotics [81]. It is also becoming increasingly clear that the extent to which phytochemical absorption can provide systemic antioxidant and anti-inflammatory activity is controlled, at least to some degree, by resident intestinal microbiota [82-84]. Finally, probiotics and the overall profile of the intestinal microbiota can influence tissue levels of mood-regulating minerals, such as magnesium and zinc [85,86].

As mentioned, intestinal microbiota may also have far-reaching effects related to glycemic control; our commensal gut microbes may contribute to healthy glucose tolerance. Indeed, the oral administration of *Bifidobacterium lactis*, and, in separate research, the combination of *Lactobacillus curvatus* and *Lactobacillus plantarum*, can improve fasting insulin levels and glucose turnover rates, even in the presence of a high-fat diet [87,88]. Again, the minimization of the detrimental LPS burden by beneficial microbes appears to be a central mechanism in the promotion of normal glycemic control [89]. For example, bifidobacteria and other beneficial microbes can prevent the efflux of LPS into systemic circulation, while in human beings, the administration of probiotics may diminish systemic access of gut-derived LPS and also reduce reactivity to the endotoxin [90].

Beyond direct nutritional and glycemic effects, there are other intriguing ways in which probiotics and the intestinal microbiota have been connected to the brain. When a strain of *Lactobacillus rhamnosus* is administered to healthy animals under stress, there is a reduction in anxiety and depression-like behaviors in experimental models, such as the elevated plus maze and forced swim tests. These behavioral changes were associated with alterations in the GABA system of the brain in the probiotic group, matching the known effects of antidepressant or anxiolytic chemical agents (for example, anxiolytic agents such as benzodiazepines work at GABA receptors) [91]. Importantly, the changes in behavior and brain chemistry were largely extinguished with vagotomy, suggesting direct lines of communication from gut to brain [90]. Additional research shows that *Lactobacillus helveticus* and *Bifidobacterium longum* added to animal drinking water can increase nerve cell resiliency and reduce apoptosis during conditions of experimental physiological stress [92]. Moreover, oral *Mycobacterium vaccae*, a soil-based microorganism widely distributed in nature, which can easily find its way onto edible plants, has been shown in experimental models to improve cognitive function and diminish anxiety-like behavior among animals [93].

There are also a number of studies involving mice reared in germ-free environments, the results of which seem to demonstrate a direct role of intestinal microbiota on behavior. Compared withconventional animals raised with the normal range of intestinal microbiota, these animals display the murine equivalent of what might be decreased anxiety [94-96]. Meanwhile, supplementation with *Bifidobacterium* appears to attenuate an exaggerated stress response and maintain adequate levels of the neuropeptide brain-derived neurotrophic factor (BDNF), levels of which are known to be low in depression [97]. It is also noteworthy that even mild chronic inflammation of the gastrointestinal tract can provoke anxiety and diminish BDNF production in animals [98]. Furthermore, supplementation with *Bifidobacterium* also provides systemic protection against lipid peroxidation and decreases brain monoamine oxidase activity, thereby potentially increasing intersynaptic neurotransmitter levels [99].

Rodent studies have provided compelling insights; however, they have countless shortcomings as a reflection of human microbiota, human dietary patterns, and the ultimate intertwining of these variables with complex mental health disorders. Far more convincing research, albeit very preliminary at this juncture, comes from published human studies involving probiotic administration. The first formal investigation of a probiotic and human mental outlook involved 132 otherwise healthy adults consuming *Lactobacillus casei* fermented beverage for three weeks; vs. placebo, significant improvement in mood scores were noted upon the among those with the higher baseline depressive symptoms [100]. A separate placebo-controlled pilot study, one using the same *Lactobacillus casei* probiotic (powder form), involved 39 chronic fatigue syndrome patients. After two months, depression scores remained unchanged between the groups, however Beck Anxiety Inventory scores showed significant improvements in anxiety versus placebo [101].

Michaël Messaoudi and colleagues from France evaluated a *Lactobacillus helveticus* and *Bifidobacterium longum* combination probiotic, which was orally administered for one month (*n*?=?55) in a placebo-controlled study[102]. Among the otherwise healthy adults, significant improvements in depression, anger, anxiety, and lower levels of the stress hormone cortisol versus placebo were noted. A concurrent experimental arm of the study also confirmed that the probiotic added to the dietary of rodents was effective in reducing behaviors indicative of anxiety. Messaoudi’s group performed a secondary analysis, looking specifically at those with the lowest baseline urinary free cortisol (*n*?=?25). Indeed, the results once again showed improvement with *Lactobacillus helveticus* and *Bifidobacterium longum* versus controls (particularly in somatization, depression and anger-hostility), and among this low cortisol sub-group the overall benefits in anxiety and depression were pronounced over time [103]. In addition, a study involving 44 patients with irritable bowel syndrome showed thatoral consumption of a prebiotic fiber (trans-galactooligosaccharide) significantly reduced anxiety in conjunction with marked elevations in fecal bifidobacteria levels [104].

Finally, a small placebo-controlled study involving functional magnetic resonance imaging (fMRI) has demonstrated that the one-month consumption of a fermented food containing *Bifidobacterium animalis* subsp *lactis*, *Streptococcus thermophilus*, *Lactobacillus bulgaricus*, and *Lactococcus lactis* subsp *lactis* can influence brain activity versus baseline [105]. Specifically, the researchers reported that the group who received the fermented dairy product, versus unfermented counterpart and the no-intervention controls, affected activity of brain regions that control central processing of emotion and sensation. Enthusiasm concerning this study runs high, with editorials in mainstream journals claiming that this fMRI study, ‘provides the first objective evidence that gut commensal and/or probiotic bacteria influence brain activity in healthy humans’,[106]. The study, of course, did not provide any such objective evidence concerning ingested bacteria; it was a study involving a transformed milk product, not an isolated probiotic powder. Despite attempts to keep caloric and macronutrient content equal, a fermented milk product is not the same as an unfermented milk product in only its microbiota. Within the study, there was no evidence of a change in gut microbiota profile via consumption of the fermented product; however, more importantly, the fermentation of milk significantly alters bioactive peptides and other chemicals that are well capable of influencing central nervous system function [107-110]. In short, objective evidence that ingested probiotic bacteria alone (or diet-induced shifts in commensal bacteria) can influence human brain activity has yet to be published.

## Traditional diets and microbiota

Before proceeding to make the case for a more focused investigation of fermented foods for mental health, it is important to discuss the available research on traditional dietary patterns and their ability to influence intestinal microbiota. It is becoming increasingly clear that indigenous or traditional dietary patterns are directly inclusive of many bacterium species that might be considered to have probiotic potential. Indeed, it is estimated that 35% of all lactic acid bacteria isolated from raw fruits and vegetables can survive gastric conditions [111]. The recent study on the anti-anxiety effects of the soil microorganism *Mycobacterium vaccae* in animals [93] suggests that we would do well to broaden our considerations of the classically defined beneficial microbes, that is, beyond that of exclusively the *Lactobacilli* and *Bifidobacteria* genera.

One of the first studies examining the effects of traditional diet, 30 years ago, looked at differences in the fecal microbiota of rural Japanese versus Canadian urbanites. The researchers noted higher counts of *Bifidobacterium* species and *Lactobacilli* in the rural Japanese, a group that largely maintained a traditional high-fiber diet rich in fermented foods, vegetables, and fish. The investigators used culture technique to examine the microbiota, and despite its limitations as a means to reflect the overall intestinal microbiome, there were some interesting findings. The amounts of *Clostridia* species in the Canadians were higher, and overall there was greater biodiversity (more genera and species) in the rural-dwelling Japanese [112]. As discussed later, this has been the primary finding of more sophisticated contemporary studies using DNA sequencing of stool samples, that is, there is more bacterial diversity in those consuming traditional diets. In follow-up, this research group reported on the differences in fecal microbiota among older adults residing in Tokyo versus elderly rural Japanese maintaining a high-fiber traditional diet inclusive of fermented foods. The results again showed higher numbers of *Bifidobacterium* species among the rural dwellers and lower amounts of *Clostridium* species, *Clostridium perfringens* in particular [113].

Recent DNA techniques allow for a more broad evaluation of the intestinal microbiome as mediated by diet. Researchers have shown significant differences in the fecal microbiota of Western European children versus rural African children living in an environment resembling that of our Neolithic ancestors. Overall, there were fewer potentially pathogenic bacteria, and a far more diverse range of microbes in rural Africans who maintain a traditional lifestyle and consume traditional foods [114]. It is noteworthy that a variety of fermented foods are consumed by those living in the rural African area which was studied, and there are numerous lactic acid bacteria present on plant foods within this traditional diet [115]. Separate work has uncovered distinct differences in microbial groups and their functional genes (for example, those governing metabolism of amino acids) in US urban dwellers versus villagers living in Africa and South America. Here again, the fecal microbiota of US urbanites showed far less diversity than that of villagers in these distinct regions. Diet, rather than hygiene *per se*, was reported to be the key spark for the development of intestinal microbiota structure [116]. Remarkably, investigations of highly preserved human coprolites (ancient stool samples retrieved from archeological sites) have demonstrated that their overall microbiome more closely resembles that of modern humans living in traditional rural settlements than that of the contemporary urban dweller [117].

What then, are the broad implications of loss of microbial diversity as a consequence of modernization? Detailed dietary analysis in combination with DNA sequencing of stool samples has its advantages. These techniques have allowed researchers to determine that long-term dietary patterns largely determine the main phyla of the gut microbial profile [118]. However, psychological stress or short-term dietary changes are capable of inducing species-level changes to the intestinal microbiota [119]. While the administration of singular or small groups of select beneficial microbes may not have a major impact on stable phyla, probiotic intervention studies (as discussed previously) have taught us that species-level application of microbes are not without clinical relevance. A single strain of *Lactobacillus*, one that might be carried with traditional foods, may improve overall microbial diversity [120]. The administration of a single *Bifidobacterium* strain, one among a genera commonly found in fermented dairy products, can increase the intestinal quantity of completely separate *Bifidobacterium* species, and *Lactobacilli* overall [121,122].

## The potential of fermented foods

Thus far, we have highlighted that depression and other mental health disorders are characterized by chronic, low-grade inflammation and oxidative stress. Conversely, a traditional diet rich in antioxidant, anti-inflammatory foods may confer some level of protection against depression. We have also noted that an intestinal ‘inflammatory microbiome’ appears to exist, one that may contribute to altered mood via intestinal permeability, systemic LPS burden, and even direct-to-brain microbe communication. Such an inflammatory microbiome may be facilitated, at least in part, by Western dietary habits. Research shows that high-fat or high-sugar and low nutrient-value foods are commonly consumed by those with depression, anxiety, and high levels of chronic distress [123-125], thus contributing to the likelihood of an inflammatory microbiome. Preliminary research in rodents and human beings suggests that the behavioral consequences of an inflammatory microbiome can be offset by the administration of beneficial microbes. All this leads us full circle to the ancient Neolithic vessels in asking to what extent fermented foods or beverages might contribute to mental health. We are certainly not the first to ask this question in the broad sense; in 1938, Lloyd Arnold, MD, aptly a professor of both preventive medicine and bacteriology at the University of Illinois, pondered to what extent ancient diets, fermented foods, and their effect on the ‘bacterial flora of the intra-intestinal contents’ converged to promote health [126].

Today, scientific advances allow for some answers in the direction toward the potential of fermented foods. It is well established that with traditional dietary patterns, fermentation can magnify protein quality [127] and the bioavailabity of mood-regulating B vitamins, magnesium, and zinc [128-131]. The effect of diet on intestinal microbiota may also extend to vitamin D levels [132]. However, it is also becoming clear that the *Lactobacillus* species isolated from traditional fermented foods are biologically active in other ways, for example, upon oral consumption, *Lactobacillus plantarum* strains isolated from traditional Chinese fermented foods provide strong antioxidant protection in animals [133]. Fermentation of fruit and herbal smoothies with *Lactobacillus plantarum* and other strains has been shown to preserve their polyphenolic compounds and vitamin C, and as expected, this enhances subsequent free radical scavenging activity that would otherwise be lost with storage [134]. Fermented soymilk also has a more pronounced antioxidant capacity than unfermented soymilk, and this activity is further enhanced by the synergistic application of both lactic acid bacteria and bifidobacteria together versus only *Lactobacilli* or *Bifidobacterium* strains alone [135].

The fermentation of fiber-rich components of traditional diets, such as, soy germ, wheat germ, rice bran, or breads made via traditional fermentation techniques, have been shown to produce novel bioactive compounds capable of producing beneficial immune, glycemic, and anti-inflammatory activities [136-139]. In the case of fermented rice bran, where enhanced phenolic availability has been noted, there is specific experimental evidence indicating beneficial mental properties via the bioactive compounds. More specifically, compared with controls, oral administration of fermented rice bran extract reduced experimental fatigue and stress [140,141]. Fermentation of rice bran, and other traditional foods, such as mung beans, buckwheat sprouts, and lentils, is known to increase the available GABA content significantly [142-144]. Whereas synthetic GABA in oral form has been classically dismissed as of limited benefit due to absorption issues, research has reported value of the oral administration of GABA derived from *Lactobacillus hilgardii* fermentation in anxiety reduction in human beings, and antidepressant activity via the administration of GABA-rich red yeast rice [145,146].

Recent evidence suggests that the health-promoting target of flavonoids is directed toward the human gut bacterial metagenomes, and that these benefits have an evolutionary origin. Functional analysis using clusters of orthologous groups of bacteria target proteins suggests that flavonoids regulate the metabolism of gut microbiota [147]. Experimental research has shown that when common dietary polyphenols are subjected to fermentation, the newly formed biotransformation phytochemicals are more capable of causing a beneficial shift in microbial growth stimulation [148]. In positioning fermented foods as worthy of discussion for cognitive and mental benefits, recent comparative research involving fermented and non-fermented foods and herbal ingredients is worthy of consideration. Researchers have examined the *in vivo* properties of an herbal blend typically used in traditional medicine to treat inflammatory disorders, comparing its effects in the unfermented and fermented form. Blood LPS levels were significantly lower when treated with the fermented blend, as was C-reactive protein, a primary marker of systemic inflammation. There was also a significant reduction in LPS-induced intestinal permeability and a significant rise in stool *Lactobacillus* species, neither of which was noted with the unfermented blend [149].

Similar research has been documented with fermented and unfermented herbs used for gastrointestinal disorders, that is, more pronounced anti-inflammatory activity and minimization of LPS-induced gene expression with the fermented blend [150]. In separate work involving a singular traditional food or medicinal agent, the anti-inflammatory botanical *Sophora flavescens,* researchers, again using LPS as the inducing agent, found a more pronounced anti-inflammatory and antioxidant activity with the fermented form [151]. Recently red wine has been shown to increase *Bifidobacterium* levels, which in turn correlates with lower serum LPS concentrations [152]. Fermented grape pomace yields more total antioxidants and antioxidant activity than its unfermented counterpart [153]. Remarkably, even fermented fish oil, an agent with well-documented anti-inflammatory and mood-support properties, has been shown to provide an enhanced anti-inflammatory activity versus its unfermented counterpart [154]. Given our previous discussions concerning LPS-induced inflammation, intestinal permeability and glycemic control, it should not be surprising, perhaps, that fermented (versus unfermented) dairy products can improve glucose metabolism and improve antioxidant status in human beings [155,156].

The connection between fermented dairy products and the growth of beneficial intestinal microbes has been well described. However, the findings that (non-dairy) fermented foods and herbs can have a positive influence on the intestinal microbiota are important in that there may be an influence on longer-term gut-brain communication. For example, isomalto-oligosaccharides are found in traditional foods (for example, honey, sake, miso, and soy sauce) and have been shown in animals and human beings to have a beneficial effect in promoting the growth of *Bifidobacteria* and *Lactobacilli*[157]. Providing just a few examples, fermented (versus unfermented) burdock has been shown to significantly promote the growth of bifidobacteria [158]; similar findings have been reported for fermented versus unfermented soy [159]. The species-level microbiota within local fermented foods is reflected in stool samples of the human host [160]. When researchers make discoveries such as that showing that a *Lactobacillus pentosus* strain derived from fermented cabbage (kimchi) can improve mental functioning and hippocampal BDNF production in animals [161], the entire mosaic takes on greater meaning. It suggests that we are only scratching the surface in our understanding of the relationship between potentially beneficial food-derived microbes and brain health.

## Conclusions

The purposeful application of fermentation for food preservation, palatability, and other reasons is an ancient art. Modern research is highlighting the potential value of ancestral dietary practices on mental health, and on resiliency against depression in particular. At the same time, there has been tremendous progress toward better understanding of the role played by the low-grade inflammation and the intestinal microbiome in human health and mental well-being [162,163]. Evidence would suggest that the two major themes of these mostly separate highways of research should converge; in other words, the fermented foods so often included in traditional dietary practices have the potential to influence brain health by virtue of the microbial action that has been applied to the food or beverage, and by the ways in which the fermented food or beverage directly influences our own microbiota. This could manifest, behaviorally, via magnified antioxidant and anti-inflammatory activity, reduction of intestinal permeability and the detrimental effects of LPS, improved glycemic control, positive influence on nutritional status (and therefore neurotransmission and neuropeptide production), direct production of GABA, and other bioactive chemicals, as well as a direct role in gut-to-brain communication via a beneficial shift in the intestinal microbiota itself.

In this discussion, we may unwittingly give the impression that fermentation is exclusively a beneficial application to food and beverage production. Such is not the case and not all forms of fermentation or fermented foods can be painted with the same brush. For example, certain microorganisms (for example, fungi) associated with pickled foods may enhance the production of *N*-nitroso compounds with potential carcinogenic properties [164]. Also, although agmatine and other polyamines found in fermented meats, fish, and certain beverages have been shown to have a variety of experimental benefits related to brain health [165,166], a safe level of intake remains unknown [165]. However, as outlined in our review, there is more than ample justification to follow the microbe-nutrition and gut-brain research pathways into convergence. The clinical world of mental health involves one where consumption of convenient, high-fat, or high-sugar foods is the norm; these foods, at odds with our evolutionary past, are not only undermining optimal nutritional status, they have untold effects on the microbiome and ultimately the brain. Hopefully, further research will continue to illuminate the ways in which the clay fermentation pots of our ancestors might be connected to the emerging discipline of nutritional psychiatry.

## Abbreviations

BDNF: brain-derived neurotrophic factor; fMRI: functional magnetic resonance imaging; GABA: gamma-aminobutyric acid; LPS: lipopolysaccharide endotoxin.

## Competing interests

ACL has received consulting fees from Genuine Health, Toronto, Canada. EMS and ACB have no competing interests.

## Authors’ contributions

All authors contributed equal time and effort to the investigation, research, and drafting of this manuscript. All authors read and approved the final manuscript.
